# Cost-Effectiveness of Liquid Biopsy for Colorectal Cancer Screening in Patients Who Are Unscreened

**DOI:** 10.1001/jamanetworkopen.2023.43392

**Published:** 2023-11-16

**Authors:** Zainab Aziz, Sophie Wagner, Alice Agyekum, Yoanna S. Pumpalova, Matthew Prest, Francesca Lim, Sheila Rustgi, Fay Kastrinos, William M. Grady, Chin Hur

**Affiliations:** 1 Department of Medicine, Columbia University Irving Medical Center, New York, New York; 2 Fred Hutchinson Cancer Center, Seattle, Washington

## Abstract

**Importance:**

Despite recommendations for universal screening, adherence to colorectal cancer screening in the US is approximately 60%. Liquid biopsy tests are in development for cancer early detection, but it is unclear whether they are cost-effective for colorectal cancer screening.

**Objective:**

To estimate the cost-effectiveness of liquid biopsy for colorectal cancer screening in the US.

**Design, Setting, and Participants:**

In this economic evaluation, a Markov model was developed to compare no screening and 5 colorectal cancer screening strategies: colonoscopy, liquid biopsy, liquid biopsy following nonadherence to colonoscopy, stool DNA, and fecal immunochemical test. Adherence to first-line screening with colonoscopy, stool DNA, or fecal immunochemical test was assumed to be 60.6%, and adherence for liquid biopsy was assumed to be 100%. For colonoscopy, stool DNA, and fecal immunochemical test, patients who did not adhere to testing were not offered other screening. In colonoscopy–liquid biopsy hybrid, liquid biopsy was second-line screening for those who deferred colonoscopy. Scenario analyses were performed to include the possibility of polyp detection for liquid biopsy.

**Exposures:**

No screening, colonoscopy, fecal immunochemical test, stool DNA, liquid biopsy, and colonoscopy–liquid biopsy hybrid screening.

**Main Outcomes and Measures:**

Model outcomes included life expectancy, total cost, and incremental cost-effectiveness ratios. A strategy was considered cost-effective if it had an incremental cost-effectiveness ratio less than the US willingness-to-pay threshold of $100 000 per life-year gained.

**Results:**

This study used a simulated cohort of patients aged 45 years with average risk of colorectal cancer. In the base case, colonoscopy was the preferred, or cost-effective, strategy with an incremental cost-effectiveness ratio of $28 071 per life-year gained. Colonoscopy–liquid biopsy hybrid had the greatest gain in life-years gained but had an incremental cost-effectiveness ratio of $377 538. Colonoscopy–liquid biopsy hybrid had a greater gain in life-years if liquid biopsy could detect polyps but remained too costly.

**Conclusions and Relevance:**

In this economic evaluation of liquid biopsy for colorectal cancer screening, colonoscopy was a cost-effective strategy for colorectal cancer screening in the general population, and the inclusion of liquid biopsy as a first- or second-line screening strategy was not cost-effective at its current cost and screening performance. Liquid biopsy tests for colorectal cancer screening may become cost-effective if their cost is substantially lowered.

## Key Points

**Question:**

Is liquid biopsy testing a cost-effective method for colorectal cancer screening, particularly in patients who refuse traditional screening methods?

**Findings:**

In this economic evaluation using a simulated cohort of patients aged 45 years with average risk of colorectal cancer, colonoscopy was the preferred (most cost-effective) screening method, with an incremental cost-effectiveness ratio of $28 071 per life-year gained. Offering liquid biopsy testing to patients who refused colonoscopy resulted in the greatest gain of life-years but was not cost-effective.

**Meaning:**

These findings suggest that in patients who refuse traditional screening for colorectal cancer, liquid biopsy is not cost-effective as an alternative screening method.

## Teaser

This economic evaluation estimates the cost-effectiveness of liquid biopsy for colorectal screening in the US.

## Introduction

Colorectal cancer (CRC) is the third most common cancer^1^ and second most common cause of cancer deaths in the US.^2^ However, there are robust screening methods for identifying both cancerous and precancerous colorectal lesions.^3^ Despite decades of evidence-based recommendations for CRC screening and precancerous polyp surveillance,^4^ screening uptake remains poor. Currently, recommended CRC screening modalities include noninvasive tests, such as fecal immunochemical test (FIT) and stool DNA, and invasive tests, such as colonoscopy.^3,5,6^ Of these, colonoscopy has the best performance for both cancer and adenoma detection, and is the most used CRC screening modality in the US.^7^ As of 2020, according to the American Cancer Society (ACS) and US Centers for Disease Control and Prevention (CDC), the average adherence to colonoscopy in US populations aged 50 to 75 years is only 60.6%.^8^ When stool tests are offered after colonoscopy is refused, the incremental improvement in screening uptake is modest, with a cumulative adherence of only 67%.^8^ This rate remains below the goal of 80% national adherence to CRC screening set by the National Colorectal Cancer Roundtable and ACS.^9^ Notably, CRCs that develop in unscreened patients are estimated to account for 28% to 44% of CRC deaths.^10^

Currently there are no blood tests recommended for CRC screening. New blood tests or liquid biopsies (LBs) using circulating tumor DNA–based markers are in development for single cancer and multicancer early detection (MCED), including CRC.^11,12,13,14^ These tests have better performance than previous blood-based cancer detection tests,^15^ and LBs may present a more appealing CRC screening option especially among individuals who are unscreened.^16^ While there has been increasing investment in LB for its potential to detect early cancer, it remains unclear whether it is a cost-effective CRC screening strategy in the US.

The purpose of this study was to estimate the cost-effectiveness of LB as a first- or second-line CRC screening strategy in the US compared with no screening and 3 approved screening strategies colonoscopy, FIT, and stool DNA. Our hypothesis was that LB would improve CRC detection and decrease the number of CRC deaths.

## Methods

This study is not considered human participants research at Columbia University; therefore, institutional review board approval was not sought, and informed consent was not needed. Reporting of this study is consistent with the Consolidated Health Economic Evaluation Reporting Standards (CHEERS) reporting guideline.

### Model Overview

**Figure 1.**
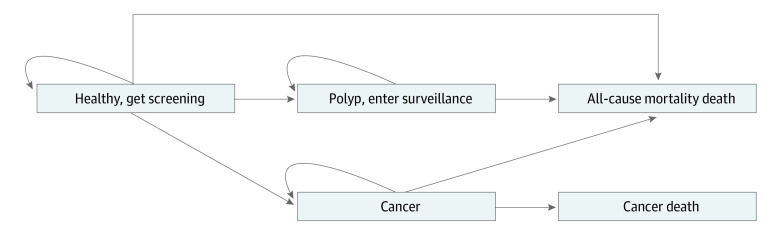
Markov Model Schematic All patients began in the Markov model healthy at age 45 years, with average risk of developing colorectal cancer. If patients were not screened, as in the natural history, there was no polyp detection or polyp surveillance, and the cancer state was entered only after diagnostic colonoscopy due to symptomatic presentation. For all other strategies, screening could detect either polyps or cancer. Death was determined by age-specific all-cause mortality, or age and stage-specific cancer death.

We created a state-transition, cohort level Markov model to assess the cost-effectiveness of LB for CRC screening compared with no screening and currently accepted screening strategies (Figure 1). The model simulated a hypothetical cohort of patients with average CRC risk in the US with a starting age of 45 years, aligning with the newest US Preventive Services Task Force (USPSTF) recommendation for CRC screening.^17^ Patients with average risk of developing CRC were not screened after age 75 years. We assumed that patients with colonic polyps were above average risk and continued polyp surveillance until age 85 years.^18^ The model had an annual cycle length, and patients were followed up until death or age 100 years. Cancer incidence and mortality data were obtained from Surveillance, Epidemiology, and End Results (SEER) registries.^19,20^ Baseline CRC incidence was derived using SEER data prior to 2000 to reflect a CRC incidence rate prior to screening guideline implementation. CRC mortality was estimated from post-2000 SEER data to reflect the most recent advances in CRC treatment. All-cause mortality rates were derived using CDC life tables.^21^ Base-case first-line screening adherence for our hypothetical cohort was 60.6% based on data from the CDC.^8^

### Strategies

The strategies analyzed were screening with colonoscopy, FIT, stool DNA (S-DNA), LB, colonoscopy-LB screening hybrid (C-LB), and no screening or natural history (NH) (eFigure 1 in Supplement 1). In the NH strategy, patients did not undergo any CRC screening. The only CRCs accounted for were those found via diagnostic colonoscopy due to symptomatic presentation. In colonoscopy, FIT, and S-DNA strategies, 60.6% of the population underwent screening while 39.4% were modeled to not adhere to screening, akin to NH.^8^ For LB, we assumed patients would agree to receive a blood test for cancer screening and adherence was set to 100%; this assumption was varied broadly in sensitivity analysis.^16^ In the C-LB group, 60.6% of patients received colonoscopy, and all unscreened patients underwent LB. Patients at average risk received colonoscopy every 10 years, S-DNA and LB every 3 years, and FIT annually. We included the possibility of complications due to colonoscopy,^22^ but did not include complications from stool tests or blood tests.

### Test Performance Characteristics

Base case estimates for sensitivity and specificity of CRC and polyp detection by colonoscopy, FIT, and stool DNA were obtained from previously published literature^23,24,25,26,27^ (Table 1). Several investigators are in the process of developing LB tests for cancer early detection, but few have detailed, published results available for analysis. For base case estimates for LB, we used data published by GRAIL for their MCED test Galleri,^11^ and extracted sensitivity and specificity for CRC detection only. This study had no data on precancerous lesions; thus, in our base case scenario, LB had no ability to detect polyps. In sensitivity and scenario analyses, we varied the performance of LB to account for the many tests in development.^28^

**Table 1.** Markov Model Inputs

Test performance characteristics	Base case	Range	Distribution	Source
**Colonoscopy^a^**				
Cancer detection rate	92.8	95% CI	Beta	Cooper et al,^26^ 2012
Interval cancer Rate	7.2	95% CI	Beta	Cooper et al,^26^ 2012
High-risk polyp sensitivity	Age specific	95% CI	Beta	Lieberman et al,^30^ 2008
Low-risk polyp sensitivity	Age specific	95% CI	Beta	Lieberman et al,^30^ 2008; Lowenfels et al,^31^ 2011
Complications	Age specific	95% CI	Beta	Knudsen et al,^23^ 2012
**FIT^a^**				
Cancer sensitivity	73.8	95% CI	Beta	Knudsen et al,^28^ 2016
High-risk polyp sensitivity	23.8	95% CI	Beta	Knudsen et al,^28^ 2016
Low-risk polyp sensitivity	7.6	95% CI	Beta	Knudsen et al,^28^ 2016
Specificity	96.4	95% CI	Beta	Knudsen et al,^28^ 2016
**Stool DNA^a^**				
Cancer sensitivity	92.3	95% CI	Beta	Knudsen et al,^28^ 2016
High-risk polyp sensitivity	42.4	95% CI	Beta	Knudsen et al,^28^ 2016
Low-risk polyp sensitivity	17.2	95% CI	Beta	Knudsen et al,^28^ 2016
Specificity	89.8	95% CI	Beta	Knudsen et al,^28^ 2016
**Liquid biopsy**				
Cancer sensitivity	82.0	70-90	Beta	Klein et al,^11^ 2021
Specificity	99.5	Not varied	Beta	Klein et al,^11^ 2021
**Costs, 2022 US $**				
Colonoscopy with polypectomy	1366	1024 to 1707	Gamma	Lansdorp-Vogelaar et al,^27^ 2010
Colonoscopy without polypectomy	1119	839 to 1399	Gamma	Lansdorp-Vogelaar et al,^27^ 2010
Fecal immunochemical test	52	39 to 65	Gamma	Lansdorp-Vogelaar et al,^27^ 2010
Stool DNA test	492	369 to 615	Gamma	Lansdorp-Vogelaar et al,^27^ 2010
Liquid biopsy test	949	0 to 949	Gamma	GRAIL website,^59^ 2023
First year costs of local cancer	42 347	31 760 to 52 934	Gamma	Lang et al,^35^ 2009
Continuing costs of local cancer	3941	2956 to 4926	Gamma	Lang et al,^35^ 2009
Final year costs of local cancer	14 158	10 618 to 17 697	Gamma	Lang et al,^35^ 2009
First year costs of regional cancer	56 019	42 014 to 70 024	Gamma	Lang et al,^35^ 2009
Continuing costs of regional cancer	8188	6141 to 10 235	Gamma	Lang et al,^35^ 2009
Final year costs of regional cancer	25 270	18 952 to 31 587	Gamma	Lang et al,^35^ 2009
First year costs of distant cancer	55 705	41 779 to 69 632	Gamma	Lang et al,^35^ 2009
Continuing costs of distant cancer	27 586	20 689 to 34 482	Gamma	Lang et al,^35^ 2009
Final year costs of distant cancer	34 464	25 848 to 43 080	Gamma	Lang et al,^35^ 2009
Cost of colonoscopy complication	Age specific	95% CI	Gamma	Knudsen et al,^23^ 2012

Abbreviation: FIT, fecal immunochemical test.

^a^ Model inputs for colonoscopy, FIT, and stool DNA were applied to natural history incidence data and therefore vary by age. Sensitivity analysis was done around the 95% CI of the table mean.

### Polyps

Data about national polyp prevalence by age group were obtained from the Clinical Outcomes Research Initiative (CORI) database.^29,30^ Polyps were defined as high risk (HR) or low risk (LR). Patients with polyps 10 mm or greater in size or with villous or high-grade dysplasia on histology were considered to have a HR polyp; patients with polyps less than 10 mm without villous or high-grade dysplasia on histology were considered to have a LR polyp.^31^ Once a polyp was diagnosed, patients underwent surveillance colonoscopy every 3 years for HR polyps and every 5 years for LR polyps until age 85 years.^32,33^

### Effectiveness and Costs

Effectiveness for each screening strategy was assessed through life-years gained (LYG) after age 45 years, indicating the average number of years a patient would live under each screening group. This modeling analysis was performed from a societal cost perspective and therefore included direct health care use costs and indirect costs, such as time lost from work. Medicare inflation-adjusted costs for colonoscopy, colonoscopy with polypectomy, FIT, and stool DNA were estimated from a previously published analysis (Table 1).^26^ Once a patient was diagnosed with CRC, costs associated with CRC treatment and living with cancer were applied. These costs were obtained from SEER-Medicare estimates and divided into first year of living with cancer, continuing cancer costs, and last year of living before death due to cancer.^34^ All costs were adjusted to 2022 US dollars using the Consumer Price Index for health care costs.^35^ Costs and LYG were discounted at an annual rate of 3%. Base-case cost of LB was approximated as the current market price of the MCED test Galleri.^36^

The primary end points were LYG, total costs in 2022 US dollars, and incremental cost-effectiveness ratios (ICERs) (eMethods in Supplement 1). We used the commonly accepted US willingness-to-pay (WTP) threshold of $100 000/LYG to determine cost-effectiveness. Secondary end points included total CRCs diagnosed and overall death due to CRC.

### Statistical Analyses

All analyses were performed using TreeAge Pro Healthcare Version 2022 R2.1 (TreeAge software). To determine the impact of model input uncertainty on cost-effectiveness results, we performed 1-way and probabilistic sensitivity analyses. In the scenario analyses, we considered cases where LB had sensitivity for HR polyps and LR polyps, adjusted LR polyp surveillance intervals to 7 and 10 years, and extended screening end age to 85 years. HR polyp sensitivity was varied between 10% and 100%, and LR polyp detection sensitivity was varied between 5% and 50%.^28^

In 1-way sensitivity analyses, model parameters are varied 1 at a time around the mean value, holding all other parameters constant. Upper and lower bounds for parameters were determined from 95% CIs around the parameter mean. For parameters where the lower bound yielded a negative value, the lower bound was set to 0. For most costs, we set the upper and lower bounds to 25% above and 25% below the mean cost, respectively. To determine at what cost LB became cost-effective compared with other strategies, we set the upper bound to its current estimated cost ($949), and the lower bound to $0.

In a probabilistic sensitivity analysis, each model input is sampled simultaneously from a probability distribution. The mean values for these distributions were base case values, and SDs were determined from literature or the corresponding database. Gamma distributions were used for costs and β distributions for all other variables.^37,38,39^ Probabilistic sensitivity analyses were performed over 5000 iterations for each strategy and the scenario where LB included polyp sensitivity.

## Results

### Base Case Results

In the NH, or no intervention strategy, 5.2% of the population developed CRC and 1.6% died from CRC, which is consistent with SEER CRC incidence from earlier decades^20^ (Table 2; eFigure 2 in Supplement 1). The population in the NH had 35.57 LYG after age 45 years with a mean cost per person of $6283. Colonoscopy was the most cost-effective screening strategy with an ICER of $28 071. It was associated with a mean cost of $9037 per person and 35.67 LYG. FIT and S-DNA strategies were both dominated by colonoscopy, meaning fewer life-years were gained compared with colonoscopy (FIT, 35.62 LYG; S-DNA, 35.64 LYG), though FIT was less expensive at $8223 and S-DNA more expensive at $11 583. C-LB had the greatest gain in life-years (35.68 LYG) and decreased total cancers and cancer deaths compared with colonoscopy. However, it was not cost-effective due to its cost of $12 006, yielding an ICER of $377 538 per LYG, above the WTP threshold of $100 000 per LYG. LB was dominated by other strategies and had a price of $15 562, though a higher proportion of cancers were diagnosed at an early stage compared with NH (eTable 1 in Supplement 1).

**Table 2.** Base Case Results

Strategy	Cost, $	LYG	Incremental cost, $	Incremental LYG	ICER, $/LYG^a^	Total cancer, %^b^	Deaths due to cancer, %^b^
Natural history	6284	35.574	[Referenc]	[Reference]	[Reference]	5.2	1.6
FIT	8223	35.624	1939	0.050	Extendedly dominated^c^	4.2	1.2
Colonoscopy	9037	35.672	2753	0.098	28 071	3.2	1.0
Stool DNA	11 583	35.641	2546	−0.031	Strictly dominated^d^	3.7	1.1
Colonoscopy–LB	12 006	35.680	2969	0.008	377 538	3.0	0.9
LB	15 562	35.581	3555	−0.099	Strictly dominated^d^	5.1	1.5

Abbreviations: ICER, incremental cost-effectiveness ratio; FIT, fecal immunochemical test; LB, liquid biopsy; LYG, life-years gained.

^a^ ICER is calculated relative to the next least costly, nondominated strategy.

^b^ Total cancer and deaths due to cancer represent cancer incidence and mortality of the entire population.

^b^ Extendedly dominated indicates that the strategy resulted in fewer LYG at a higher cost per life-year gained compared with another strategy.

^d^ Strictly dominated indicates that the strategy resulted in higher costs and fewer LYG compared with another strategy.

### Scenario Analyses

**Figure 2.**
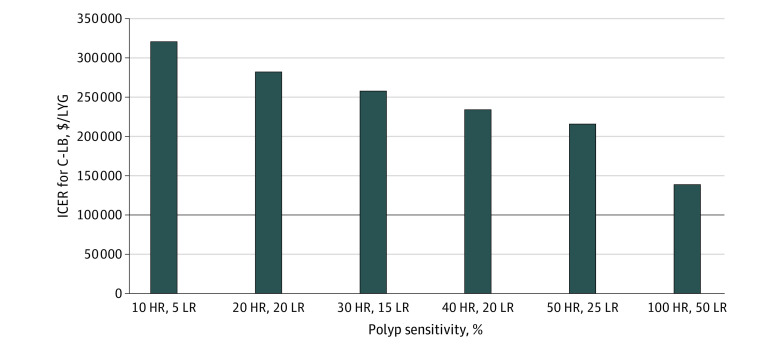
Scenario Analysis for LB Polyp Detection in C-LB Incremental cost-effectiveness ratios (ICERs) associated with varying liquid biopsy (LB) polyp sensitivity in the colonoscopy-LB (C-LB) screening group. The black line indicates an ICER of $100 000 per life-year gained (LYG), or the US willingness-to-pay threshold. At its current price, even with polyp sensitivity as high as 100% sensitivity for high-risk polyps and 50% sensitivity for low risk polyps, C-LB ICER compared with traditional screening strategies does not reach the US willingness-to-pay threshold. HR indicates high-risk polyp; LR, low-risk polyp.

We varied the ability of LB to detect polyps over a range of sensitivities (Figure 2; eTable 2 in Supplement 1). At 10% HR polyp sensitivity and 5% LR polyp sensitivity, C-LB screening had more LYG than the base case (0.009 LYG). The ICER decreased to $319 834 per LYG but was still above the WTP threshold and was therefore not cost-effective. LB remained dominated, though LYG increased to 35.60 from 35.58 base case. C-LB and LB remained not cost-effective even with polyp sensitivity as high as 50% to 100%. Scenario analyses adjusting number of years between LR polyp screening (eTable 3 in Supplement 1), and end age of screening (eTable 4 in Supplement 1) were also done. Neither analysis differed from base-case model conclusions.

### One-Way Sensitivity Analyses

**Figure 3.**
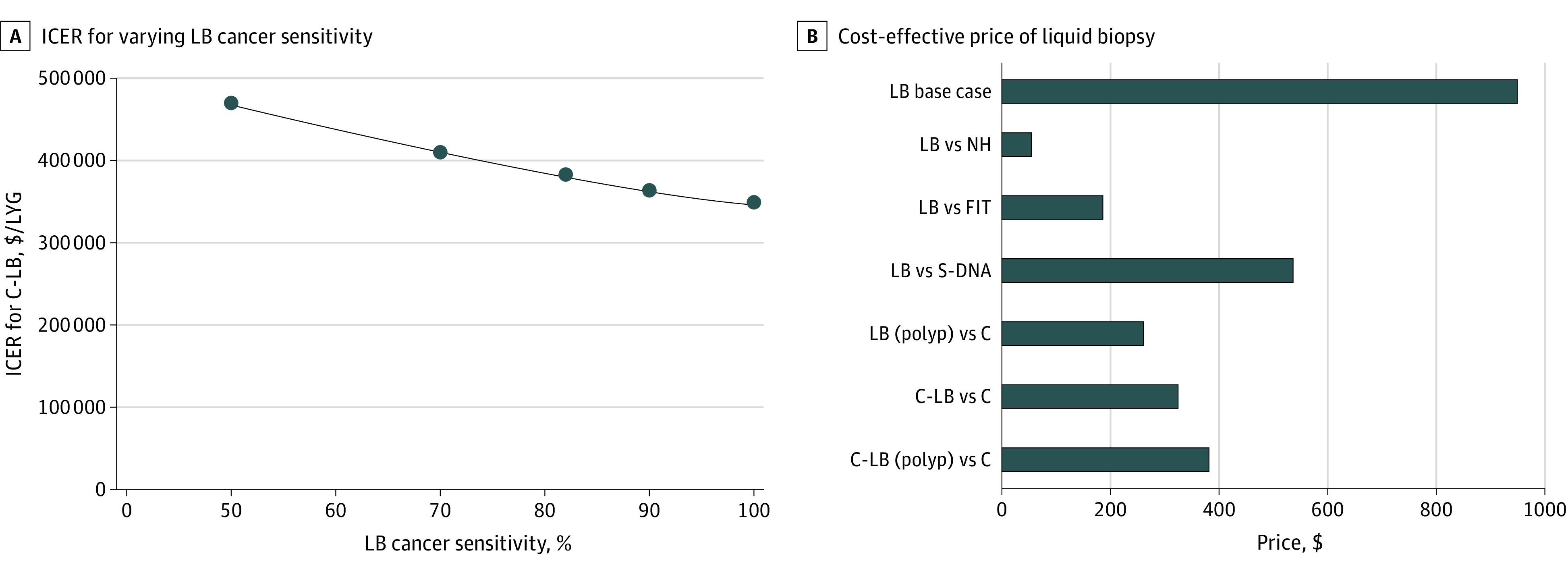
Sensitivity Analyses for Liquid Biopsy (LB) Cancer Sensitivity and Cost A, Incremental cost-effectiveness ratios (ICERs) associated with varying liquid biopsy (LB) cancer sensitivity in the colonscopy-LB (C-LB) screening arm. The base case sensitivity for LB cancer detection was 82%. When cancer sensitivity for LB was varied over a range of 50% to 100%, the ICER for LB remained dominated (not pictured) and the ICER for C-LB remained above the willingness-to-pay threshold of $100 000 per life-year gained (LYG), represented by the black line. ICERs are calculated relative to traditional screening strategies included in base case results. B, Analysis for cost-effective price of LB. The base case price of LB was set at $949, as shown in the top bar. Cost-effective price of LB compared with other strategies was determined via 1-way sensitivity analysis. LB (polyp) and C-LB (polyp) represent scenarios where LB included polyp sensitivity, with high-risk polyp sensitivity at 20% and low risk polyp sensitivity at 10%. FIT indicates fecal immunochemical test; NH, natural history; S-DNA, stool DNA.

Results from 1-way sensitivity analyses were compared between 2 strategies. We compared NH with LB; colonoscopy with LB with polyp sensitivity (20% HR polyp sensitivity; 10% LR polyp sensitivity); and colonoscopy with C-LB with polyp sensitivity. We also directly compared the cost of LB with all other strategies (Figure 3).

Comparing NH with LB, the model was most sensitive to the cost of LB testing, followed by the performance characteristics of LB. The cost of LB had to be decreased to $56.16 for it to be cost-effective with an ICER below the $100 000 per LYG WTP threshold. When comparing colonoscopy with LB with polyp sensitivity, the parameters the model was most sensitive to were the cost of LB, followed by cost of colonoscopy and sensitivity of LB for polyps. Cost of LB had to be decreased to $260.95 for it to become cost-effective. Lastly, when comparing colonoscopy with C-LB with polyp sensitivity, the model was most sensitive to the performance of LB detection for cancer, LB cost, and the ability for LB to detect polyps. The cost of LB had to be reduced to $382.10 for C-LB to become cost-effective. We performed further sensitivity analyses by adjusting performance estimates and adherence rates for LB. The base case test sensitivity for cancer was 82% (range, 50%-100%), based on data from literature. We varied this sensitivity between 50% and 100%. Across the range of values for this parameter, total cancers and cancer deaths for LB and for C-LB remained stable. Overall cost of the screening strategies changed minimally and ICERs remained over a $100 000 per LYG WTP threshold in the case of C-LB, or dominated in the case of LB. Reductions in LB adherence led to decreased costs but increased cancer burden and cancer deaths for both LB and C-LB. Summary of 1-way sensitivity analyses is included in Figure 3 and eTables 5 and 6 in Supplement 1.

### Probabilistic Sensitivity Analyses

Using a WTP threshold of $100 000 per LYG, colonoscopy was the cost-effective strategy in 86.8% of the 5000 iterations, C-LB hybrid was cost-effective in 12.2% of iterations, FIT was cost-effective in 1% of iterations, and stool DNA and LB were the cost-effective strategy in less than 1% of iterations (eFigure 3 in Supplement 1). In the scenario where LB testing included polyp sensitivity, with a mean sensitivity of 20% for HR polyp detection and 10% for LR polyp detection, colonoscopy remained the most cost-effective screening strategy 96.9% of the time at a WTP of $100 000 per LYG (eFigure 4 in Supplement 1).

## Discussion

In this study, we used a Markov simulation to analyze the cost-effectiveness of LB, used both as a novel first or second-line screening modality. We present the first analysis that integrates novel LB into paradigms for CRC screening and systematically explores scenarios to determine the cost-effectiveness of LB.

The most cost-effective screening strategy in our base-case model was colonoscopy, with an ICER of $28 071 per LYG. While C-LB had the highest number of LYG and prevented the most cancers, the cost of LB would have to reduce by 66% (from $949 to $324) for the C-LB strategy to become cost-effective in our model. Compared with NH, the cost of LB would have to be reduced by 94% for its ICER to drop below the WTP threshold of $100 000 per LYG. When compared with stool-based tests, the cost of LB would have to decrease by 43% to 80% to be cost-effective. LB and C-LB had more LYG when polyp detection was introduced, but they did not achieve cost-effectiveness at LB’s current price even with perfect performance.

Adherence to CRC screening recommendations continues to be a significant public health concern. Patients cite many reasons for not getting CRC screened, including distress with bowel preparations, discomfort handling stool samples, and difficulty taking time off work.^40,41,42,43^ Given the numerous barriers, there is tremendous opportunity to engage unscreened patients. LB is far easier to complete than stool tests or colonoscopy, because it can be done alongside routine bloodwork. Our LB strategy modeled whether offering LB to an unscreened population would be cost-effective compared with no screening.

Prior analyses have studied the cost-effectiveness of CRC screening strategies,^44,45^ including less sensitive blood-based biomarkers.^15^ Our analysis focuses on a new category of blood-based screening tests that could have better performance with the advent of efficient next-generation sequencing and application of artificial intelligence for cancer prediction. Other studies have analyzed the use of LB for risk assessment of patients living with advanced cancers.^46,47,48^ To our knowledge, this is the first study that also considers novel LB in a cost-effectiveness analysis for CRC screening.

While LB is not currently widely available for routine clinical use, it will likely become easy to request by physician prescription.^49^ Many LB tests are in preparation for FDA approval, and more than one test has been awarded breakthrough device designation as recently as January of 2023, including one for MCED (pancreatic, ovarian, esophageal, liver, and lung cancer).^50^ With multiple LB tests soon to enter the market, and a potential reduction in price pending FDA approval, conducting a cost-effectiveness analysis is timely. Our analysis provides threshold targets for LB performance and cost to guide future test development and clinical and policy decision making.

### Limitations

This study has limitations. In the absence of observed data to compare our results against, we calibrated our model to historical SEER data and cross-validated our results against previous CRC screening models. We made the assumption that patients would be 100% adherent to any follow-up testing. This included follow-up colonoscopy after a positive stool or LB test, or long-term polyp surveillance. Incorporating a patient’s prior behavior and allowing it to affect future choices would have greatly increased the complexity of our model and results. To address this, we performed sensitivity analysis on overall adherence rates.

Base case sensitivity and cost for LB testing was limited to the results of a single peer-reviewed study. Other LB companies have shared data in press releases and abstracts.^28,51,52^ Differences in their test characteristics are accounted for in sensitivity analyses for LB cancer detection and scenario analyses for polyp detection. We similarly varied cost in sensitivity analysis as cost of LB will likely decrease with advancing technology. We did not adjust specificity, because this was already set at 99.5%, and adjusting any lower would worsen the performance of LB. While many LB tests are CRC specific, others are marketed as MCED tests.^53^ Our analysis only took into consideration the cost-effectiveness for CRC screening; future analyses could take into account the cumulative result of screening for multiple cancers at once. We did not include the possible harms from LB testing, such as patient anxiety due to false-positive results.^54,55,56^

Our model approximates CRC incidence, mortality, and screening adherence for the total US population. The model does not account for racial and ethnic variation, thereby mitigating the known increased cancer mortality among people who belong to minoritized racial and ethnic groups.^57,58^ We do not believe the outcomes of this study would significantly change if the analyses were done for separate racial and ethnic groups because LB was only marginally more effective than no screening.

## Conclusions

In this economic evaluation of LB for CRC screening in the US, screening with LB was not cost-effective compared with current screening strategies. Furthermore, for individuals who refused traditional screening, it was not cost-effective to offer LB over no screening. LB tests for CRC screening may become cost-effective in the future if they are significantly less expensive or if polyp detection is introduced coupled with a decrease in cost.

## Notes

Supplement 1. **eFigure 1. **CRC Screening Strategies Considered **eMethods. **Evaluating Cost-Effectiveness **eTable 1. **Cancer Stage Distribution by Screening Strategy **eFigure 2. **Cost-Effectiveness Plane **eTable 2. **Liquid Biopsy Polyp Sensitivity Scenario Analyses Clinical End Points **eTable 3. **Scenario Analyses for Low Risk Polyp Surveillance Interval **eTable 4. **Scenario Analyses For Screening End Age **eTable 5. **Sensitivity Analysis for Liquid Biopsy Adherence **eTable 6. **Sensitivity Analysis for Liquid Biopsy Cancer Sensitivity and Cost **eFigure 3. **Probabilistic Sensitivity Analysis for Base-Case Screening Strategies **eFigure 4. **Probabilistic Sensitivity Analysis for Liquid Biopsy with Polyp Detection

Supplement 2.
Data Sharing Statement
