# Supplementary material for: Cost-Effectiveness of Liquid Biopsy for Colorectal Cancer Screening in Patients Who Are Unscreened
Source: JAMA Netw Open. 2023 Nov 16;6(11):e2343392. doi: 10.1001/jamanetworkopen.2023.43392 (PMC10654798; doi:10.1001/jamanetworkopen.2023.43392)
Supplement: Supplement 1. — eFigure 1. CRC Screening Strategies Considered eMethods. Evaluating Cost-Effectiveness eTable 1. Cancer Stage Distribution by Screening Strategy eFigure 2. Cost-Effectiveness Plane eTable 2. Liquid Biopsy Polyp Sensitivity Scenario Analyses Clinical End Points eTable 3. Scenario Analyses for Low Risk Polyp Surveillance Interval eTable 4. Scenario Analyses For Screening End Age eTable 5. Sensitivity Analysis for Liquid Biopsy Adherence eTable 6. Sensitivity Analysis for Liquid Biopsy Cancer Sensitivity and Cost eFigure 3. Probabilistic Sensitivity Analysis for Base-Case Screening Strategies eFigure 4. Probabilistic Sensitivity Analysis for Liquid Biopsy with Polyp Detection [file jamanetwopen-e2343392-s001.pdf]

Supplemental Online Content

Aziz Z, Wagner S, Agyekum A, et al. Cost-effectiveness of liquid biopsy for colorectal cancer screening in patients who are unscreened. *JAMA Netw Open*. 2023;6(11):e2343392. doi:10.1001/jamanetworkopen.2023.43392

eFigure 1. CRC Screening Strategies Considered
eMethods. Evaluating Cost-Effectiveness
eTable 1. Cancer Stage Distribution by Screening Strategy
eFigure 2. Cost-Effectiveness Plane
eTable 2. Liquid Biopsy Polyp Sensitivity Scenario Analyses Clinical Endpoints
eTable 3. Scenario Analyses for Low Risk Polyp Surveillance Interval
eTable 4. Scenario Analyses for Screening End Age
eTable 5. Sensitivity Analysis for Liquid Biopsy Adherence
eTable 6. Sensitivity Analysis for Liquid Biopsy Cancer Sensitivity and Cost
eFigure 3. Probabilistic Sensitivity Analysis for Base-Case Screening Strategies
eFigure 4. Probabilistic Sensitivity Analysis for Liquid Biopsy With Polyp Detection

This supplemental material has been provided by the authors to give readers additional information about their work.

© 2023 Aziz W et al. *JAMA Network Open.*

eFigure 1. CRC Screening Strategies Considered

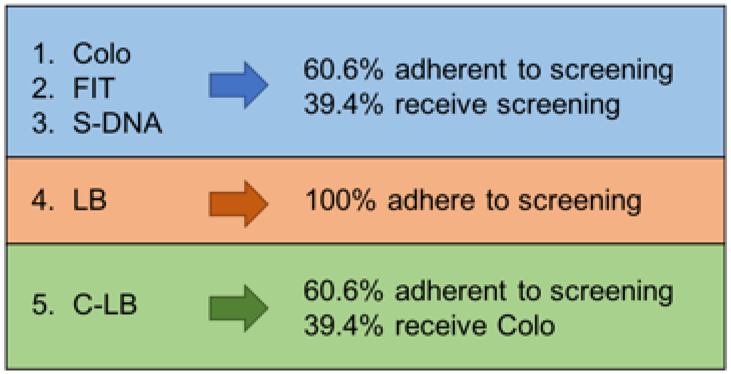

Abbreviations: Colo, colonoscopy; FIT, fecal immunochemical test; S-DNA, stool DNA; LB, liquid biopsy; C-LB, colonoscopy-liquid biopsy hybrid

© 2023 Aziz W et al. *JAMA Network Open.*

eMethods

Evaluating Cost-Effectiveness

An incremental cost-effectiveness ratio (ICER) is a commonly used measure in cost-effectiveness analyses that represents the economic value of a certain intervention compared with another intervention. More specifically, it is the additional cost of a 1 unit increase of an outcome for a specific strategy when compared with another strategy. In our analysis, our outcome of interest is Life-Years Gained (LYG). LYG are used to measure health outcomes in terms of the number of years lived. The calculation of an ICER is shown below:

$$ICER = \frac{\Delta Cost}{\Delta LYG}$$

When we are comparing multiple interventions simultaneously, interventions are ordered from lowest to highest cost. Strictly dominated interventions have fewer LYG and higher costs compared with another intervention. These interventions are removed from any consideration and do not have a calculated ICER. Once all strictly dominated interventions are removed, ICERs are calculated for each strategy in comparison with its next lower cost alternative. This means no ICER is calculated for the strategy that has the lowest cost. If a strategy has an ICER greater than the next more costly alternative, it is extendedly dominated which means it results in fewer LYG with a greater cost per LYG than its comparator. Extendedly dominated strategies are removed from consideration and do not have a calculated ICER. All ICERs are recalculated after the removal of extended dominated strategies. Both strictly dominated and extendedly dominated strategies do not represent an efficient use of sources and are not considered cost-effective when compared with other included strategies.

To determine the cost-effectiveness of an intervention, we compare the intervention's ICER with a willingness-to-pay threshold (WTP) which represents the maximum cost a health system is willing to pay for a benefit in health. If the ICER is below the WTP threshold, the intervention is cost-effective compared with its reference strategy. If the ICER is below the WTP threshold, the intervention is not cost-effective compared with its reference strategy.

© 2023 Aziz W et al. *JAMA Network Open.*

eTable 1. Cancer Stage Distribution by Screening Strategy

Strategy	Local Cancer (%)	Regional Cancer (%)	Distant Cancer (%)
No Screening	2.3	2.0	0.9
FIT	2.1	1.5	0.6
Colonoscopy	1.5	1.2	0.5
Stool DNA	1.7	1.4	0.6
Colonoscopy-LB	1.5	1.0	0.5
Liquid Biopsy	2.5	1.8	0.8

Abbreviations: FIT, fecal immunochemical test; LB, liquid biopsy

Percent of population under each screening strategy that develops local, regional, or distant cancer.

© 2023 Aziz W et al. *JAMA Network Open.*

eFigure 2. Cost-Effectiveness Plane

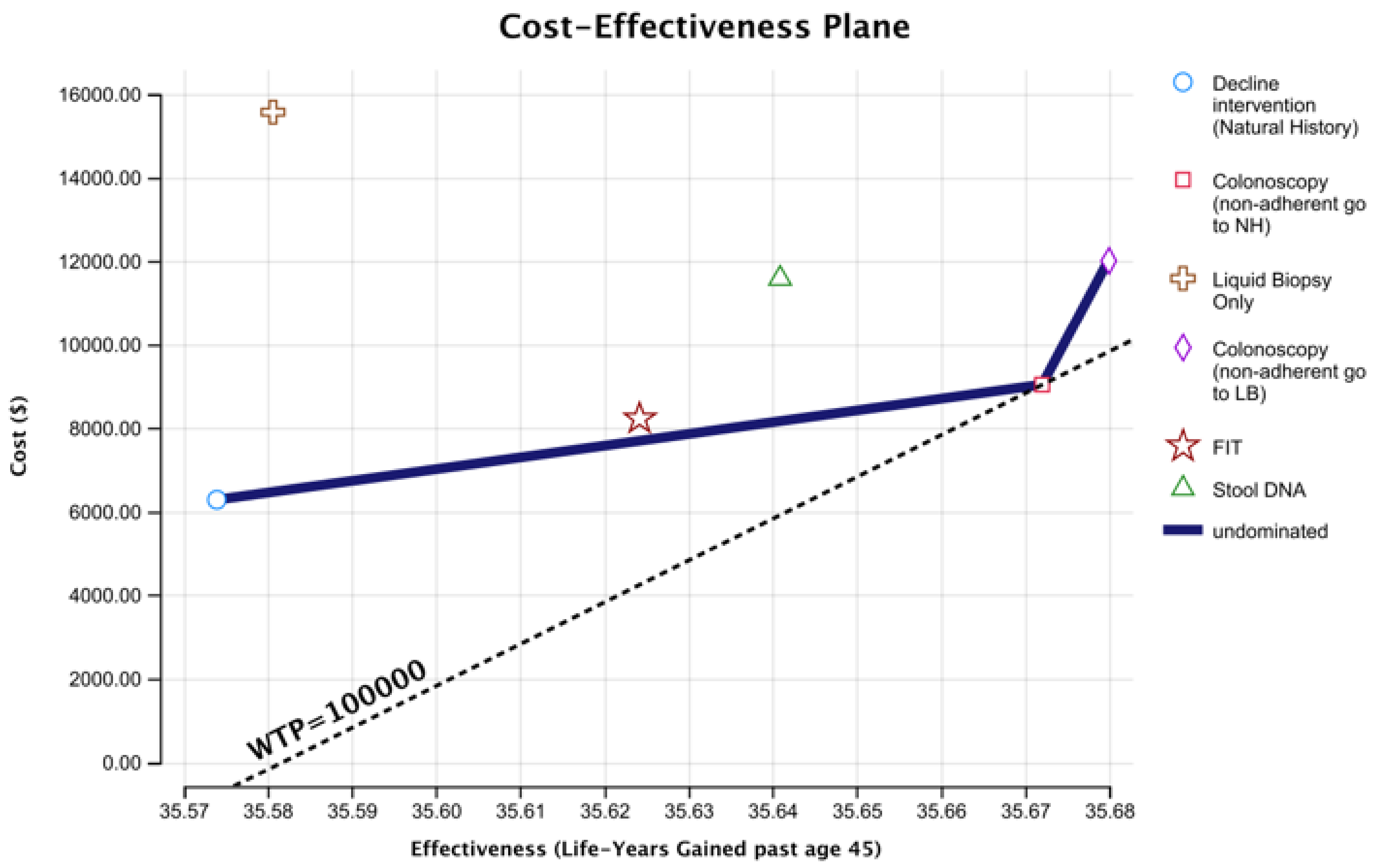

Abbreviations: NH, natural history; FIT, fecal immunochemical test; LB, liquid biopsy; WTP, willingness-to-pay

Solid line represents the efficiency frontier. Any strategy that is above the efficiency frontier is not cost-effective compared to strategies that exist on the efficiency frontier.

© 2023 Aziz W et al. *JAMA Network Open.*

eTable 2. Liquid Biopsy Polyp Sensitivity Scenario Analyses Clinical Endpoints

	Total Cancer (%)[a]	Deaths due to Cancer (%)[a]	Cost ($)	LYG	ICER[b] ($/LYG)
10% HR polyp sensitivity, 5% LR polyp sensitivity					
LB	4.1	1.2	22,112	35.598	Strictly Dominated[c]
C-LB	2.7	0.7	14,336	35.689	319,834
20% HR polyp sensitivity, 10% LR polyp sensitivity					
LB	4.1	1.2	21,786	35.601	Strictly Dominated[c]
C-LB	2.5	0.7	14,167	35.690	282,245
30% HR polyp sensitivity, 15% LR polyp sensitivity					
LB	4.0	1.1	21,405	35.607	Strictly Dominated[c]
C-LB	2.5	0.7	14,097	35.692	258,336
40% HR polyp sensitivity, 20% LR polyp sensitivity					
LB	3.9	1.1	21,051	35.611	Strictly Dominated[c]
C-LB	2.5	0.7	13,977	35.693	234,184
50% HR polyp sensitivity, 25% LR polyp sensitivity					
LB	3.8	1.0	20,690	35.615	Strictly Dominated[c]
C-LB	2.5	0.7	13,860	35.694	216,061
100% HR polyp sensitivity, 50% LR polyp sensitivity					
LB	3.4	1.0	18,930	35.639	Strictly Dominated[c]
C-LB	2.4	0.6	13,259	35.702	140,036

Abbreviations: LYG, life-years gained; ICER, incremental cost-effectiveness ratio; HR, high risk; LR, low risk; LB, liquid biopsy; C-LB, colonoscopy-liquid biopsy hybrid
[a]Total Cancer (%) and Deaths Due to Cancer (%) represent cancer incidence and mortality of the entire population.
[b]ICER is calculated relative to the next least costly, non-dominated strategy from the base-case strategies.
[c]Strictly Dominated indicates that the strategy resulted in higher costs and fewer life-years gained compared with another strategy.

© 2023 Aziz W et al. *JAMA Network Open.*

eTable 3. Scenario Analyses for Low Risk Polyp Surveillance Interval

Strategy	Cost ($)	LYG	Incremental Cost ($)	Incremental LYG	ICER ($/LYG)[a]	Total Cancer (%)[d]	Deaths due to Cancer (%)[d]
LR polyp surveillance every 7 years							
**Natural History**	6,284	35.574	Reference	Reference	Reference	5.2	1.6
FIT	8,080	35.617	1,797	0.043	Extendedly Dominated[b]	4.3	1.2
**Colonoscopy**	9,031	35.657	2,747	0.083	32,997	3.5	1.0
Stool DNA	11,449	35.634	2,418	-0.023	Strictly Dominated[c]	3.8	1.1
**Colonoscopy-LB**	11,995	35.665	2,964	0.008	389,236	3.3	1.0
Liquid Biopsy	15,551	35.580	3,556	-0.085	Strictly Dominated[c]	5.2	1.6
LR polyp surveillance every 10 years							
**Natural History**	6,284	35.574	Reference	Reference	Reference	5.2	1.6
FIT	7,899	35.612	1,615	0.038	Extendedly Dominated[b]	4.4	1.3
**Colonoscopy**	8,600	35.647	2,317	0.073	31,585	3.7	1.1
Stool DNA	11,269	35.630	2,669	-0.018	Strictly Dominated[c]	3.9	1.2
**Colonoscopy-LB**	11,559	35.655	2,958	0.007	396,908	3.5	1.1
Liquid Biopsy	15,533	35.580	3,974	-0.075	Strictly Dominated[c]	5.2	1.6

Abbreviations: LYG, life-years gained; ICER, incremental cost-effectiveness ratio; FIT, fecal immunochemical test; LB, liquid biopsy
[a]ICER is calculated relative to the next least costly, non-dominated strategy
[b]Extendedly dominated indicates that the strategy resulted in fewer life-years gained at a higher cost per life-year gained compared with another strategy
[c]Strictly dominated indicates that the strategy resulted in higher costs and fewer life-years gained compared with another strategy
[d]Total Cancer (%) and Deaths due to Cancer (%) represent cancer incidence and mortality of the entire population

Increasing length of surveillance interval for patients found to have a low risk polyp does not affect the conclusions of the cost-effectiveness analysis results. Colonoscopy remains the most cost-effective strategy, with Liquid Biopsy and Stool-DNA strictly dominated, and FIT extendedly dominated. While Colonoscopy-LB is not dominated, its ICER remains above the willingness-to-pay threshold of $100,000, meaning it is not a cost-effective strategy.

© 2023 Aziz W et al. *JAMA Network Open.*

eTable 4. Scenario Analyses For Screening End Age

Strategy	Cost ($)	LYG	Incremental Cost ($)	Incremental LYG	ICER ($/LYG)[a]	Total Cancer (%)[d]	Deaths due to Cancer (%)[d]
All strategies end screening at 85							
**Natural History**	6,284	35.574	Reference	Reference	Reference	5.2	1.6
FIT	8,275	35.625	1,991	0.051	Extendedly Dominated[b]	4.1	1.1
**Colonoscopy**	8,958	35.676	2,674	0.102	26,210	3.0	0.9
Stool DNA	11,705	35.646	2,747	-0.030	Strictly Dominated[c]	3.5	1.1
**Colonoscopy-LB**	12,827	35.681	3,869	0.005	801,525	2.9	0.9
Liquid Biopsy	16,907	35.584	4,081	-0.097	Strictly Dominated[c]	5.0	1.5
LB ends screening at 85; all other end screening at 75							
**Natural History**	6,284	35.574	Reference	Reference	Reference	5.2	1.6
FIT	8,223	35.624	1,939	0.050	Extendedly Dominated[b]	4.2	1.2
**Colonoscopy**	9037	35.672	2,753	0.098	28,071	3.2	1.0
Stool DNA	11,583	35.641	2,546	-0.031	Strictly Dominated[c]	3.7	1.1
**Colonoscopy-LB**	12,913	35.677	3,876	0.005	802,986	2.9	0.9
Liquid Biopsy	16,907	35.584	3,994	-0.093	Strictly Dominated[c]	5.0	1.5

Abbreviations: LYG, life-years gained; ICER, incremental cost-effectiveness ratio; FIT, fecal immunochemical test; LB, liquid biopsy
[a]ICER is calculated relative to the next least costly, non-dominated strategy
[b]Extendedly dominated indicates that the strategy resulted in fewer life-years gained at a higher cost per life-year gained compared with another strategy
[c]Strictly dominated indicates that the strategy resulted in higher costs and fewer life-years gained compared with another strategy
[d]Total Cancer (%) and Deaths due to Cancer (%) represent cancer incidence and mortality of the entire population

Ending screening at age 85, rather than 75, does not change the conclusions of the analysis. Colonoscopy remains the most cost-effective strategy, and the ICER of Colonoscopy-LB increases. Overall the cost of all strategies, other than Colonoscopy, increases for increasing age of screening, an expected increase. The model predicts that the cost of an additional colonoscopy is less costly than the cost of treating cancers discovered between the ages of 75 and 85 as a result of no screening. We hypothesize that the model does not account for the true cost of regular, screening colonoscopy at an older age for patients of average risk, including increased complications, societal costs, etc. Despite this discrepancy, the model conclusions remain unchanged, and Liquid Biopsy and Colonoscopy-LB remain not cost-effective.

© 2023 Aziz W et al. *JAMA Network Open.*

eTable 5. Sensitivity Analysis for Liquid Biopsy Adherence

Total Cancer (%)[a]	Deaths due to Cancer (%)[a]	Cost ($)	LYG	ICER ($/LYG)[b]
60.6% adherence to LB in LB arm				
5.2	1.6	11,906	35.57804	Dominated[c]
19.7% of patients receive LB in C-LB arm				
3.1	0.9	10,410	35.676	349,135
6.4% of patients receive LB in C-LB arm				
3.1	0.9	9,333	35.67335	231,085

Abbreviations: LYG, life-years gained; ICER, incremental cost-effectiveness ratio; LB, liquid biopsy; C-LB, colonoscopy-liquid biopsy hybrid
[a]Total Cancer (%) and Deaths Due to Cancer (%) represent cancer incidence and mortality of the entire population.
[b]ICER is calculated relative to the next least costly, non-dominated strategy from the base-case strategies.
[c]Dominated indicates that the strategy resulted in higher costs and fewer life-years gained compared with another strategy.

60.6% adherence to LB in the LB arm is equivalent to adherence rates set in the base-case analysis for Colo, FIT, and S-DNA. Half of patients who refuse colonoscopy in the C-LB arm is equivalent to 19.7% of the population. “19.7% of patients receive LB in C-LB arm” represents a scenario where half of unscreened patients remain unscreened. Similarly, “6.4% of patients receive LB in C-LB arm” represents a scenario where only 6.4% of unscreened patients receive LB, akin to the rate of stool testing uptake following refusal of colonoscopy in the US.

© 2023 Aziz W et al. *JAMA Network Open.*

eTable 6. Sensitivity Analyses for Liquid Biopsy Cancer Sensitivity and Cost

Sensitivity Analysis for Liquid Biopsy Cost	
Comparator	Cost-effective price ($)
Liquid Biopsy vs Natural History	56.16
Liquid Biopsy vs FIT	187.76
Liquid Biopsy vs Stool DNA	536.31
Liquid Biopsy (polyp) vs Colonoscopy	260.95
Colonoscopy-LB vs Colonoscopy	324.82
Colonoscopy-LB (polyp) vs Colonoscopy	382.10
Sensitivity Analysis for Liquid Biopsy cancer sensitivity	
Liquid Biopsy Sensitivity	ICER[a]
50% cancer sensitivity	
LB	Dominated[b]
C-LB	465,299
70% cancer sensitivity	
LB	Dominated[b]
C-LB	406,350
90% cancer sensitivity	
LB	Dominated[b]
C-LB	360,408
100% cancer sensitivity	
LB	Dominated[b]
C-LB	341,043

Abbreviations: FIT, fecal immunochemical test; LB, liquid biopsy; ICER, incremental cost-effectiveness ratio
[b]ICER is calculated relative to the next least costly, non-dominated strategy from the base-case strategies.
[c]Dominated indicates that the strategy resulted in higher costs and fewer life-years gained compared with another strategy.

Both with and without ability to detect polyps, liquid biopsy became cost-effective (ICER below willingness-to-pay threshold of $100,000/life-years gained) compared to other strategies and C-LB became cost effective compared to Colo when LB's cost was reduced. Polyp detection was set at 20% sensitivity for high risk polyps and 10% sensitivity for low risk polyps. Neither LB or C-LB became cost effective when test sensitivity for cancer was varied.

© 2023 Aziz W et al. *JAMA Network Open.*

eFigure 3. Probabilistic Sensitivity Analysis for Base-Case Screening Strategies

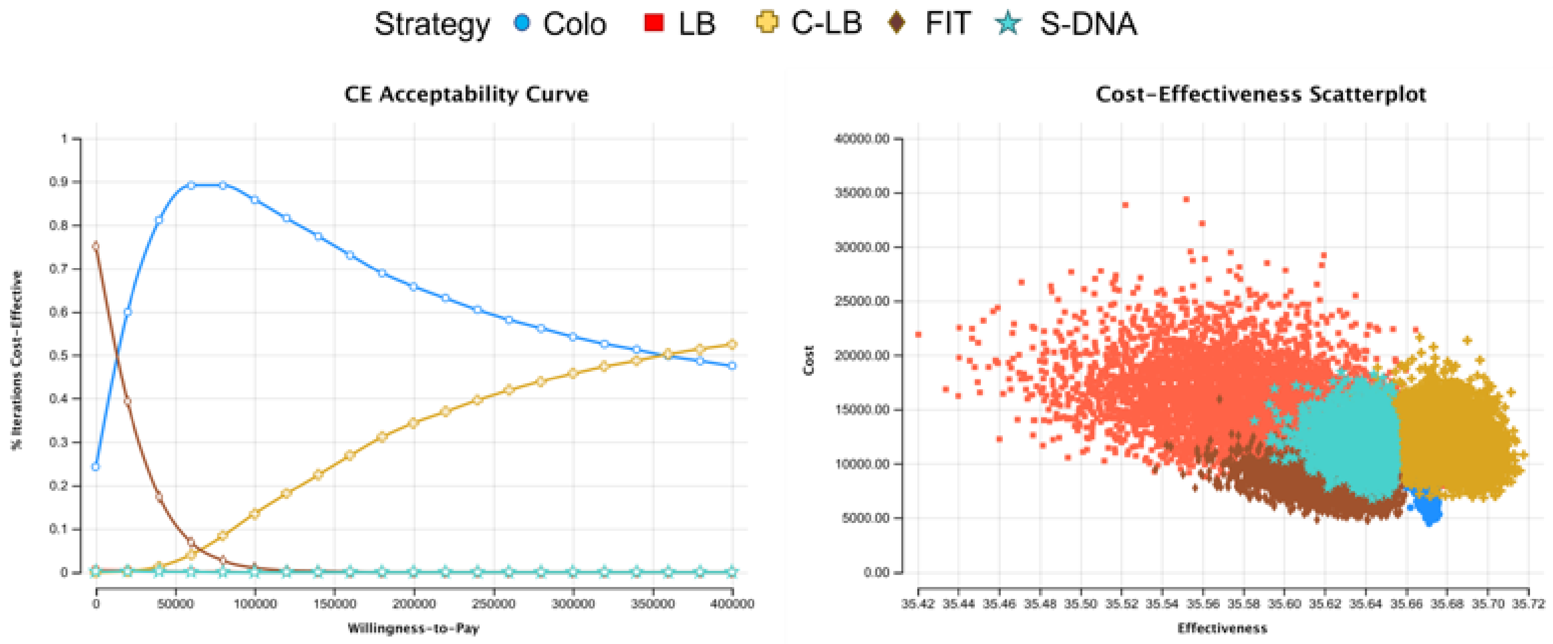

Abbreviations: CE, cost-effectiveness; Colo, colonoscopy; FIT, fecal immunochemical test; S-DNA, stool DNA; LB, liquid biopsy; C-LB, colonoscopy-liquid biopsy hybrid

CE Acceptability curve shows the percent of iterations each strategy is the cost-effective strategy over a range of willingness-to-pay thresholds. The Cost-Effectiveness Scatterplot shows the costs and effectiveness over 5000 iterations for each strategy in the base case for our probabilistic sensitivity analysis.

© 2023 Aziz W et al. *JAMA Network Open.*

eFigure 4. Probabilistic Sensitivity Analysis for Liquid Biopsy with Polyp Detection

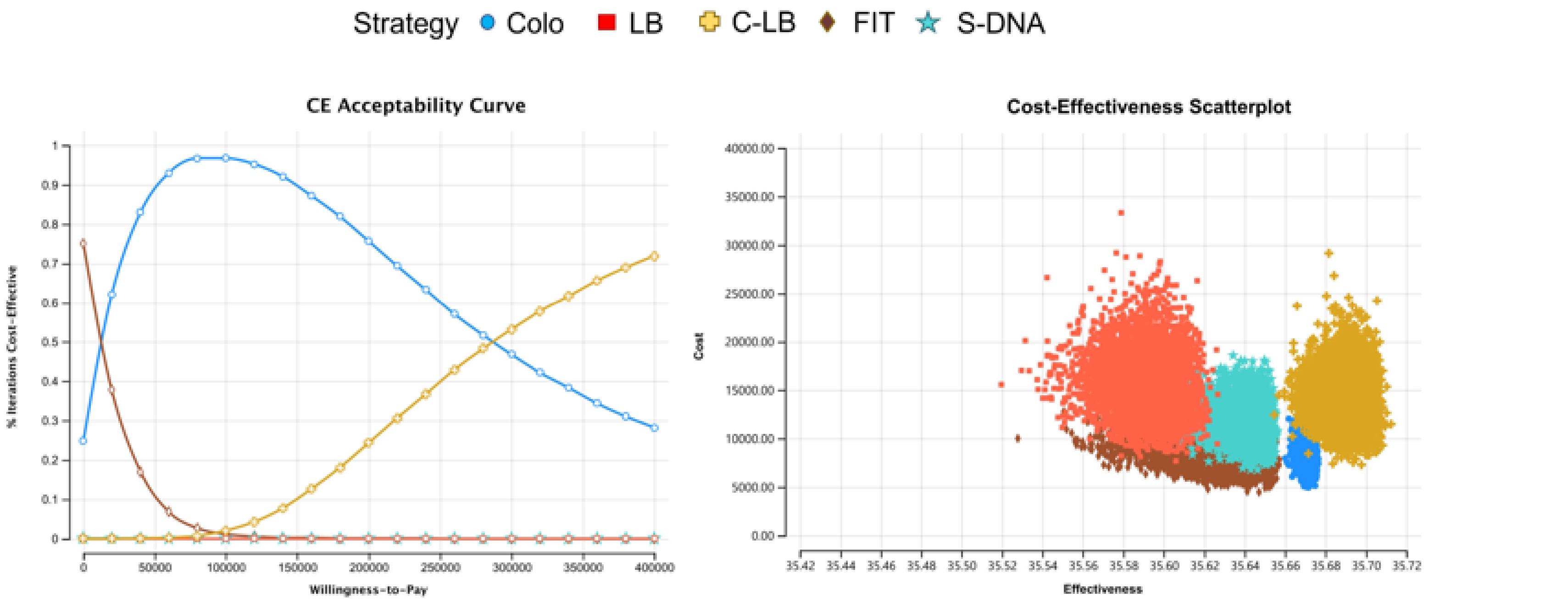

Abbreviations: CE, cost-effectiveness; Colo, colonoscopy; FIT, fecal immunochemical test; S-DNA, stool DNA; LB, liquid biopsy; C-LB, colonoscopy-liquid biopsy hybrid

CE Acceptability curve shows the percent of iterations each strategy is the cost-effective strategy over a range of willingness-to-pay thresholds. The Cost-Effectiveness Scatterplot shows the costs and effectiveness over 5000 iterations from our probabilistic sensitivity analysis. FIT, S-DNA, and Colo strategies from the base case and LB and C-LB where liquid biopsy has polyp sensitivity are included in the above analysis. Mean high risk polyp sensitivity was set at 20% and mean low risk polyp sensitivity was set at 10%.

© 2023 Aziz W et al. *JAMA Network Open.*
