# Supplementary material for: Cost-Effectiveness of Liquid Biopsy for Colorectal Cancer Screening in Patients Who Are Unscreened
Source: JAMA Netw Open. 2023 Nov 16;6(11):e2343392. doi: 10.1001/jamanetworkopen.2023.43392 (PMC10654798; doi:10.1001/jamanetworkopen.2023.43392)
Supplement: Supplement 2. — Data Sharing Statement [file jamanetwopen-e2343392-s002.pdf]

Data Sharing Statement

Data
Data available: Yes
Data types: Data (not involving human participants)
How to access data: za2278@cumc.columbia.edu
When available: With publication

Supporting Documents
Document types: None

Additional Information
Who can access the data: Researchers who would like data
Types of analyses: any purpose
Mechanisms of data availability: with investigator support
